# Dopaminergic neurons show increased low-molecular-mass protein 7 activity induced by 6-hydroxydopamine in vitro and in vivo

**DOI:** 10.1186/s40035-018-0125-9

**Published:** 2018-08-17

**Authors:** Ming-Shu Mo, Gui-Hua Li, Cong-Cong Sun, Shu-Xuan Huang, Lei Wei, Li-Min Zhang, Miao-Miao Zhou, Zhuo-Hua Wu, Wen-Yuan Guo, Xin-Ling Yang, Chao-Jun Chen, Shao-Gang Qu, Jian-Xing He, Ping-Yi Xu

**Affiliations:** 1 Department of Neurology, First Affiliated Hospital of Guangzhou Medical University, Guangzhou, 510120 Guangdong China; 2 Department of Neurology, Qilu Hospital of Shandong University, Jinan, 250012 Shandong China; 3 Department of Neurology, First Affiliated Hospital of Sun Yat-sen University, Guangzhou, 510080 Guangdong China; 4 Department of Neurology, Second Affiliated Hospital of Xinjiang Medical University, Urumchi, 830011 Xinjiang China; 5 Clinic Brain Center, Guangzhou Hospital of Integrated Traditional and Western Medicine, Guangzhou, 510800 Guangdong China; 6 Department of Blood Transfusion, Fifth Affiliated Hospital Southern Medical University, Guangzhou, 510900 Guangdong China; 7 Department of Thoracic Surgery, First Affiliated Hospital of Guangzhou Medical University, Guangzhou, 510120 Guangdong China

**Keywords:** Parkinson’s disease, 6-hydroxydopamine, Immunoproteasome, TAP1

## Abstract

**Background:**

Abnormal expression of major histocompatibility complex class I (MHC-I) is increased in dopaminergic (DA) neurons in the substantia nigra (SN) in Parkinson’s disease (PD). Low-molecular-mass protein 7 (β5i) is a proteolytic subunit of the immunoproteasome that regulates protein degradation and the MHC pathway in immune cells.

**Methods:**

In this study, we investigated the role of β5i in DA neurons using a 6-hydroxydopamine (6-OHDA) model in vitro and *vivo*.

**Results:**

We showed that 6-OHDA upregulated β5i expression in DA neurons in a concentration- and time-dependent manner. Inhibition and downregulation of β5i induced the expression of glucose-regulated protein (Bip) and exacerbated 6-OHDA neurotoxicity in DA neurons. The inhibition of β5i further promoted the activation of Caspase 3-related pathways induced by 6-OHDA*.* β5i also activated transporter associated with antigen processing 1 (TAP1) and promoted MHC-I expression on DA neurons.

**Conclusion:**

Taken together, our data suggest that β5i is activated in DA neurons under 6-OHDA treatment and may play a neuroprotective role in PD.

**Electronic supplementary material:**

The online version of this article (10.1186/s40035-018-0125-9) contains supplementary material, which is available to authorized users.

## Background

Oxidative stress, accumulation of aggregated and misfolded protein aggregates, and neuroinflammation have been suggested to play roles in the pathogenesis of Parkinson’s disease (PD) [1, 2] These factors impair the ubiquitin-proteasome system (UPS) which is critical for protein metabolic homeostasis [3–5], and they promote the replacement of constitutive proteasome subunits β1, β2 and β5 by the respective immunoproteasome catalytic subunits β1i/ low-molecular-mass protein 2 (LMP2, PSMB9), β2i/multicatalytic endo- peptidase complex-like 1 (MECL1, PSMB10) and β5i (LMP7, PSMB8) [6–8]. The immunoproteasome helps to degrade abnormal proteins, present cleaved peptides as antigens to major histocompatibility complex (MHC) molecules and regulate neuroinflammation [9, 10].

Immunoproteasome expression is low in normal young human brains but higher in brain specimens from older normal subjects and Alzheimer’s disease (AD) patients [11, 12]. LMP2 knockout mice show classic AD-like symptoms and severe oxidative stress involved in Aβ aggregation [12, 13]. In Huntington’s disease (HD), immunoproteasomes may contribute to the metabolism of huntingtin protein, which is not easily degraded by classical proteasomes [14]. β5i also plays an important role in the regulation of oxidative stress in chronic epilepsy and stroke [15, 16]. β5i expression and changes in proteasomal structure have been found in tyrosine hydroxylase (TH^+^) cells in postmortem brains of people with PD-like synucleinopathies such as multiple system atrophy (MSA) and progressive supranuclear palsy (PSP) [17]. β5i is known to shape the antigenic repertoire presented on MHC-I. A recent study demonstrated that catecholamine neurons were more responsive to MHC-I expression under γ-interferon (IFN) treatment and that these neurons were more susceptible to neurotoxicity in neuroinflammatory conditions than in control conditions [18, 19]. However, whether β5i contributes to DA neuronal neurotoxicity remains unclear. In this study, we further explored the role of β5i in the loss of dopaminergic (DA) neurons under 6-hydroxydopamine (6-OHDA) insult in vitro and *vivo*.

## Methods

### Cell culture

SN4741 cells derived from embryonic substantia nigra and maintained in Dulbecco’s-modified Eagle’s high-glucose medium (DMEM, Life Technologies, Rockville, MD, USA) supplemented with 10% fetal calf serum (FCS, Irvine Scientific, Santa Ana, CA, USA), 1% glucose (Sigma, St. Louis, MO, USA), 1% penicillin–streptomycin (Gibco™, Invitrogen, China) and 2 mmol/L l-glutamine (Gibco™, Invitrogen, China). SN4741 cells were kindly provided by Prof. Qian-Yang of the Fourth Military Medical University [20]. Cells were grown at 37 °C in 5% CO_2_ and subcultured every 3 days as described previously [21].

### Cell viability, reactive oxygen species (ROS) and chymotrypsin-like function

CCK-8 (Dojindo, Kumamoto, Japan) was used to analyze cell viability under different treatment conditions according to the manufacturer’s recommendations. Cells were trypsinized, suspended and cultured in 96-well plates at a concentration of 5× 10^3^ cells/well. Each sample was made in triplicate. The plate contained blank, positive and negative control wells. PR-957 (Selleck, Houston, CA, USA) was used as a selective inhibitor of β5i in SN4741 cells [22]. Cells were treated with 6-OHDA or PR-957 overnight or for 48 h, respectively, or with control solution. At different time points, 10 μl cell counting kit-8 (CCK-8) (Dojindo, Kumamoto, Japan) solution dissolved in 100 μl DMEM (Life Technologies, Rockville, MD, USA) replaced the drug in each well. The incubation continued for another 0.5, 1, or 2 h at 37 °C following the manufacturer’s instructions. The optical density (OD) value at 450 nm was measured to calculate cell viability using the formula: cell viability (%) = [OD (Sample)-OD (blank control)]/ [OD (negative control)-OD (blank control)] by an ELISA microplate reader (ELX800, BioTeK, USA).

Rhodamine 123 (Sigma-Aldrich, St Louis, MO, USA) was used to measure the mitochondrial membrane potential disruption. Cells were suspended and cultured in 6-well plates. After overnight incubation, groups were exposed to 6-OHDA, PR-957 or control solution. Cells were washed 3 times with PBS and reincubated with 100 μl DMEM (Life Technologies, Rockville, MD, USA) containing 10 μg/mL rhodamine 123 at 37 °C for 30 mins. The fluorescence of rhodamine 123 was detected by a fluorescence spectrophotometer (Shimadzu, Matsuyama, Japan, RF5000U) at 490 nm excitation (Ex) and 520 nm emission (Em).

2′, 7′-Dichlorofluorescin diacetate (DCFH-DA; Sigma-Aldrich, St Louis, MO, USA) was used to measure ROS level following manufacturer’s recommendations. Cells were treated with 6-OHDA or PR-957 at different concentrations and exposure durations. After the cells were washed 3 times with PBS, DCFH-DA diluted in DMEM to 10 μM was added and incubated at 37 °C for 20 min. Cells were washed 3 times with DMEM, and the resultant optical density was measured at 488 nm excitation and 525 nm emission by a microplate reader (Spectramax Gemini XS, Molecular Devices, Pennsylvania, USA). The amount of generated ROS was calculated using the formula: [OD (Sample)-OD (Negative control)]/ OD (Negative control).

The chymotrypsin-like activity (CTL) of the immunoproteasome was assayed with Suc-LLVY-AMC [2]. Cells were seeded at a concentration of 1× 10^4^ cells/well in 96-well plates. Each test was performed in 4 replicates. After treatment with different concentrations of 6-OHDA, cells were harvested and lysed in proteolysis buffer (50 mM Tris-HCl pH 7.4, 5 mM MgCl2, 1 mM DTT ± 0.25 mM ATP). Then, 100 μl containing 2 μg cell lysate was mixed with 50 μM Suc-LLVY-AMC (Sigma-Aldrich, St Louis, MO, USA). After 1 h of equilibration, fluorescence was monitored for 3 h using a SpectraMax M5 plate reader (Molecular Devices, Pennsylvania, USA, Ex/Em: 370 nm/460 nm).

### Overexpression plasmid and shRNA transfection

The β5i overexpression plasmid was synthesized by GeneCopoeia (Product ID: EX-Mm34282-M29, GeneCopoeia, Guangzhou, China). This sequence was inserted into a p-EZ-M29 vector containing neomycin as a stable selection marker. The insertion was confirmed by sequencing. The mU6 vector contained the mCherryFP gene as a marker to identify transfection efficiency (Product ID: CSHCTR001, GeneCopoeia, Guangzhou, China). PSMB8 was suppressed by specific shRNA in the mU6 vector (Product ID: RSH052242-mU6, GeneCopoeia, Guangzhou, China) with target sequences GGAATGCAGCCCACTGAATTC, GGAAGGTTCAGATTGAAATGG, GCAGGAAGTTACATTGCTACC and GCCAAGGAATGCAGGCTATAC and the hairpin loop sequence TCAAGAG. The mU6-pri vector (Product ID: CSHCTR001-mU6, GeneCopoeia, Guangzhou, China) without the target gene and an empty plasmid were used in the negative control (NC) and mock (M) groups, respectively. First, we detected β5i mRNA by qQT-PCR and then confirmed β5i protein expression by Western blot.

Transfection was performed based on manufacturer’s instructions (Invitrogen, Grand Island, NY, USA). Cells were suspended and seeded in 24-well plates at a 50% cell density after counting. After 24 h of culture, transfection was performed as follows. Solution A contained 20 pmol shRNA dissolved in 50 μl Opti-MEM without serum, and B solution contained 1 μl lipofectamine 3000 (Invitrogen, Grand Island, NY, USA) dissolved in 50 μl Opti-MEM without serum. Solution A and B were mixed and kept at room temperature for 20 min. The culture medium for each well was replaced with 400 μl serum-free medium. Cells were incubated in this mixture (serum-free medium containing solutions A and B) for 6 h for transfection, which was then replaced with serum medium. Transfection efficiency was assessed by fluorescence on the following day.

### Partial 6-OHDA lesion and behavioral test

Forty male Sprague Dawley (SD) rats, ranging from 280 to 300 g in weight, were bred and maintained in the Specific Pathogen-Free Laboratory Animal Center at Guangzhou Medical University (Guangzhou, China). Weight-matched rats were randomly assigned to four groups: the sham group, 6-OHDA (Sigma-Aldrich, St Louis, MO, USA) group, PR-957 (Adooq Bioscience, CA, USA) group and 6-OHDA plus PR-957 group. Rats were anesthetized with ketamine (10%) /xylazine (2%) (Sigma Aldrich, St Louis, MO, USA) and injected with 8 μg 6-OHDA in 4 μl solvent [0.9% *w*/*v* NaCl with 0.1% ascorbic acid (Sigma-Aldrich, St Louis, MO, USA)] into the left anterior medial bundle (Coordinates: AP: - 4.0 mm, ML: - 1.5 mm, DV: - 7.8 mm). Animals in the 6-OHDA plus PR-957 group were given the same dose of 6-OHDA followed by 4 μl PR-957 (50 nM) injected into the lateral ventricle. The sham group was given the same volume of solvent [0.9% *w*/*v* NaCl with 0.1% ascorbic acid]. At 4 weeks after the 6-OHDA injection, rats were tested in the rotation test. Rotation asymmetry was calculated for 30 min after intraperitoneal injection of 0.6 mg/kg apomorphine (Sigma-Aldrich, St Louis, MO, USA) as described previously [23]. All animal studies followed the institutional guidelines for animal experiments of Guangzhou Medical University. All procedures were approved by the Institutional Animal Care and Use Committee of Guangzhou Medical University.

### Western blot

After electrophoresis of proteins from SN4141 cells or the midbrain of rats and blocking with 0.5% BSA in PBS, the PVDF membranes (Pall Corporation, Pensacola, FL, USA) were incubated with primary antibodies such as anti-β5i (1:800, Abcam, Cambridge, MA, USA), anti-β5 (1:1000, Abcam, Cambridge, MA, USA) or anti-β-actin (1:2000, CST, Danvers, MA, USA) at 4 °C overnight. The primary antibodies were diluted in blocking solution (LI-COR Biosciences, Lincoln, NE, USA). After the membranes were washed, they were incubated with fluorescent-conjugated secondary antibodies (1: 15000; LI-COR Biosciences, Lincoln, NE, USA) for 1 h in the dark. The Odyssey infrared fluorescence detection system (LI-COR Biosciences, Lincoln, NE, USA) was used for scanning and analysis. For traditional Western blot, secondary antibodies conjugated with horseradish peroxidase (HRP, Santa Cruz Biotechnology, Santa Cruz, CA, USA) and the chemical luminescence detection method (ECL, Pierce Biotechnology, Rockford, IL, USA) were used. Data were scanned and analyzed using the GE 600 system (GE Healthcare, Piscataway, NJ, USA).

Following the protocol used by Goyal et al. [24], 1.0 × 10^4^ cells were inoculated in 96-well plates (Corning, Sigma-Aldrich, Dorset, UK) for the in-cell western assay. Cells were cultured in DMEM with 10% FCS (Irvine Scientific, Santa Ana, CA, USA) for 48 h, which was then replaced with 6-OHDA dissolved in FCS-free DMEM, but the control group was cultured in FCS-free DMEM. Then, each well was washed with PBS and fixed in 4% formaldehyde for 1 h. Formaldehyde was washed away with PBS, and cells were incubated with 0.1% Triton X-100 in PBS (3 times, 5 min each). Then, cells were treated with blocking solution (LI-COR Biosciences, Lincoln, NE, USA) and incubated with mouse anti-β5i (1:800, Abcam, Cambridge, MA, USA) and rabbit anti-β5i (1:800, Abcam, Cambridge, MA, USA) overnight at 4 °C. After the cells were washed, fluorescent-conjugated secondary antibodies (LI-COR Biosciences, Lincoln, NE, USA), diluted at 1: 1000 in PBS, were added, and the cells were incubated for 1 h in the dark at room temperature. Cells were with PBS three times in the dark. Then, plates were imaged on an Odyssey infrared scanner (LI-COR Biosciences, Lincoln, NE, USA).

### Immunofluorescence staining and immunohistochemistry

Brain tissue was cut at a thickness of 15 μm and stored at − 20 °C. Primary antibodies used for immunohistochemistry included mouse monoclonal anti-tyrosine hydroxylase (TH) (1:500, MAB318, Merck Millipore, Billerica, MA, USA), anti-β5i (1:500, Abcam, Cambridge, MA, USA) and anti-TAP-1 (1:500, ab10356; Abcam, Cambridge, MA, USA). TAP-1 is a downstream protein that receives peptides provided by the immunoproteasome [25]. After overnight incubation with primary antibodies, the tissue or cells were incubated with secondary antibodies such as Cy3-conjugated anti-mouse IgG (1:400, Jackson Immuno-research laboratory, PA, USA) and/or Alexa 488-conjugated anti-rabbit IgG (1:400, Molecular Probes, Eugene, OR, USA). Images were acquired using a fluorescence microscope (BX51, Olympus, Fujinon, Japan). For immunohistochemistry, the secondary antibody used was a horseradish peroxidase (HRP)-conjugated goat anti-mouse IgG (1:1000, Kangcheng, Shanghai, China). Sections were stained with 3, 3′-diaminobenzidine (DAB) kits (Wuhan Boster Bioengineering Co., Ltd., Wuhan, China). Images were acquired under a microscope (Olympus AX70; Olympus, Tokyo, Japan). Four images at 200× magnification were taken, with each image covering an area of the SN or striatum, and combined into one figure. Images were analyzed by ImageJ software (version 1.45; National Institutes of Health, Bethesda, Maryland, USA).

### Fast TH staining and laser capture microdissection (LCM)

To reduce RNA degradation, we used fast TH staining to detect DA neurons. Slices were fixed in acetone-methanol solution at − 20 °C for 10 min, washed with PBS containing 1% Triton X-100, incubated with the TH antibody (MAB318, Merck Millipore, Billerica, MA, USA) at a 1:100 dilution for 10 min, rinsed in PBS with Triton twice, and incubated with the goat antirabbit antibody with HRP (1:100, Kangcheng, Shanghai, China) for 5 min. Immunohistochemistry staining was done by DAB kits (Wuhan Boster Bioengineering Co., Ltd., Wuhan, China). The stained slices were dehydrated in RNase-free solutions as follows: 100% acetone for 5 min, 75% ethanol, 95 and 100% ethanol for 1 min each, and then xylene twice for 1 min and 5 min.

As described previously [26, 27], nonfixed fresh brain tissue was rapidly frozen and cut into 8-μm-thick slices. Slices were collected on to polyethylene naphthalate membrane-coated glass slides (Life Technologies, Grand Island, NY, USA). After fast tyrosine hydroxylase staining, TH^+^ neurons in the substantia nigra were captured by the Arcturus XT system (Life Technologies, CA, USA). Laser power was set at 70 mW and 150 mV. Approximately 300–450 TH^+^ neurons were collected, and total RNA was extracted using the mirVana PARIS Kit (PN AM1556, Austin, TX, Ambion, USA) and converted into cDNA by a Reverse Transcription Kit (Takara, Shiga, Japan). RT1A (rat monomorphic MHC class I antigen) binds the peptide or antigens translocated by TAP into the ER, and its mRNA level in DA neurons was detected by qRT-PCR. The PCR primers (TIANGEN Biotech, China) used were as follows: GAPDH-F: 5’-TACTAGCGGTTTTACGGGCG-3′ and GAPDH-R: 5’-TCG-AACAGGAGGAGCAGAGAGCGA-3′; TAP-1-F: 5’-GGCAGACTCAGTTC-CTCTCAC-3′ and TAP-1-R: 5’-CAGAACGGGTTGGGGATCAA-3′; RT1A (Rat monomorphic MHC class I antigen) -F: 5’-GCTCACACTCGCTGCGGTAT-3′ and RT1A-R: 5’-GCCATACATCTCCTGGATGG-3′. GAPDH was used as an internal control, and mRNA expression was analyzed using the 2^−ΔΔCT^ method [28].

### Statistical analysis

All experiments were repeated at least 3 times. Data are shown as the mean ± SD. ANOVA was followed by Tukey’s or Student-Newman-Keuls (SNK) post hoc testing. *P* < 0.05 was considered statistically significant. All analyses were performed using SPSS.13 and STATA software (Version 14; StataCorp, College Station, TX, USA).

## Results

### 6-Hydroxydopamine upregulates immunoproteasome expression in DA neurons

**Fig. 1:**
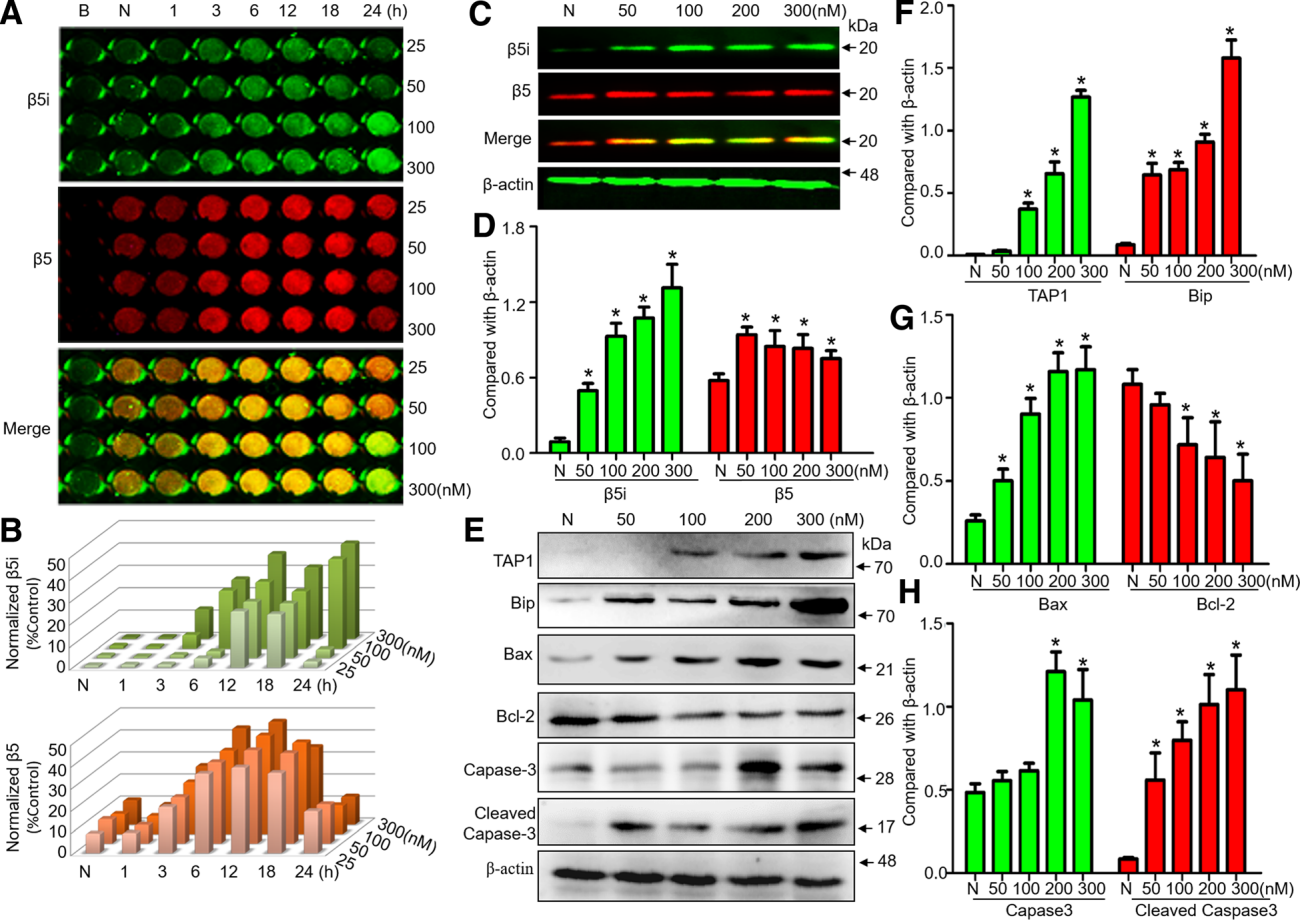
6-Hydroxydopamine activates immunoproteasomes in DA neurons in a dose-dependent manner. **a** The expression of β5 and β5i in SN4741 cells under 6-OHDA treatment by in-cell western assay and (**b**) the fluorescence intensity in each group after normalization to that in the normal group are shown. **c-h** The expression of β5, β5i, TAP1, Bip, Bax, Bcl-2, Caspase-3 and Cleaved Caspase-3 in SN4741 cells after different concentrations of 6-OHDA treatment for 24 h. Data are presented as the mean ± SD; *n* = 4 experiments; two-way ANOVA and post hoc SNK t-test

SN4741 cells were treated with different concentrations of 6-OHDA for 24 h. The in-cell western assay showed that β5 was upregulated upon treatment with 6-OHDA for 3–18 h and then downregulated after 24 h, whereas β5i was upregulated by 100–300 nM 6-OHDA for 24 h (Fig. 1a, b). The Western blot data further confirmed that β5 and β5i expression were dose-dependently upregulated when the concentration of 6-OHDA was higher than 50 nM (Fig. 1c, d). In the antigen presentation pathway, the expression of glucose-regulated protein (Bip), a regulator involved in protein translocation into the ER [29], and TAP1, a transporter associated with antigen presentation [30], was increased as a dose-dependent manner under 6-OHDA treatment for 24 h (Fig. 1e, f). In the apoptosis pathway, the expression of proapoptotic proteins, such as Bax, Caspase-3 and cleaved-Caspase-3, was upregulated, and the expression of antiapoptotic proteins, such as Bcl-2, was downregulated significantly by 6-OHDA treatment for 24 h (Fig. 1g, h).

**Fig. 2:**
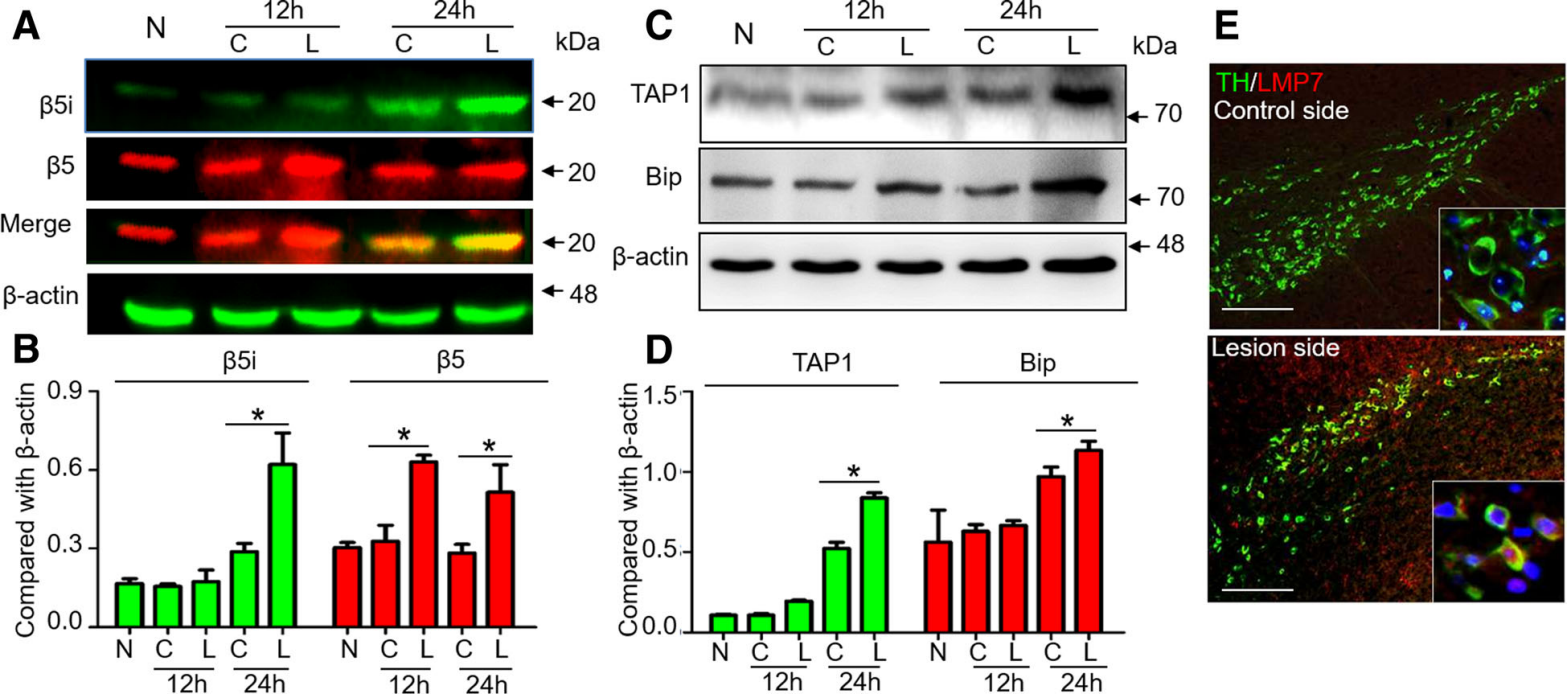
6-Hydroxydopamine activates immunoproteasomes in DA neurons in vivo. The expression of β5, β5i (**a, b**), TAP1 and Bip (**c, d**) in the SN of the 6-OHDA hemilesioned rat model. **e** The colocalization of β5i on TH neurons in the SN at 1 day after 6-OHDA treatment. Scale bar = 100 μm. * *P* < 0.05, compared with control. N, normal group without treatment. C, control side of the brain. L, lesioned side of the brain. Data are presented as the mean ± SD; *n* = 4 experiments; one-way ANOVA and post hoc SNK t-test

When SN4741 cells were treated with 200 nM 6-OHDA for different exposure times, β5 was upregulated, reaching a peak expression at 12 h (Additional file 1: Figure S1. A, B), whereas β5i, TAP1 and Bip expression were significantly increased upon 6-OHDA treatment at 24 h (Additional file 1: Figure S1. A-D). In vivo, increased expression of β5, β5i, TAP1 and Bip was further confirmed in the SN of the rat after 6-OHDA treatment for 24 h (Fig. 2a-d) and was accompanied by an increased percent of neurons with β5i and TH^+^ expression (Fig. 2e).

### β5i dysfunction inhibits antigen presentation in DA neurons

**Fig. 3:**
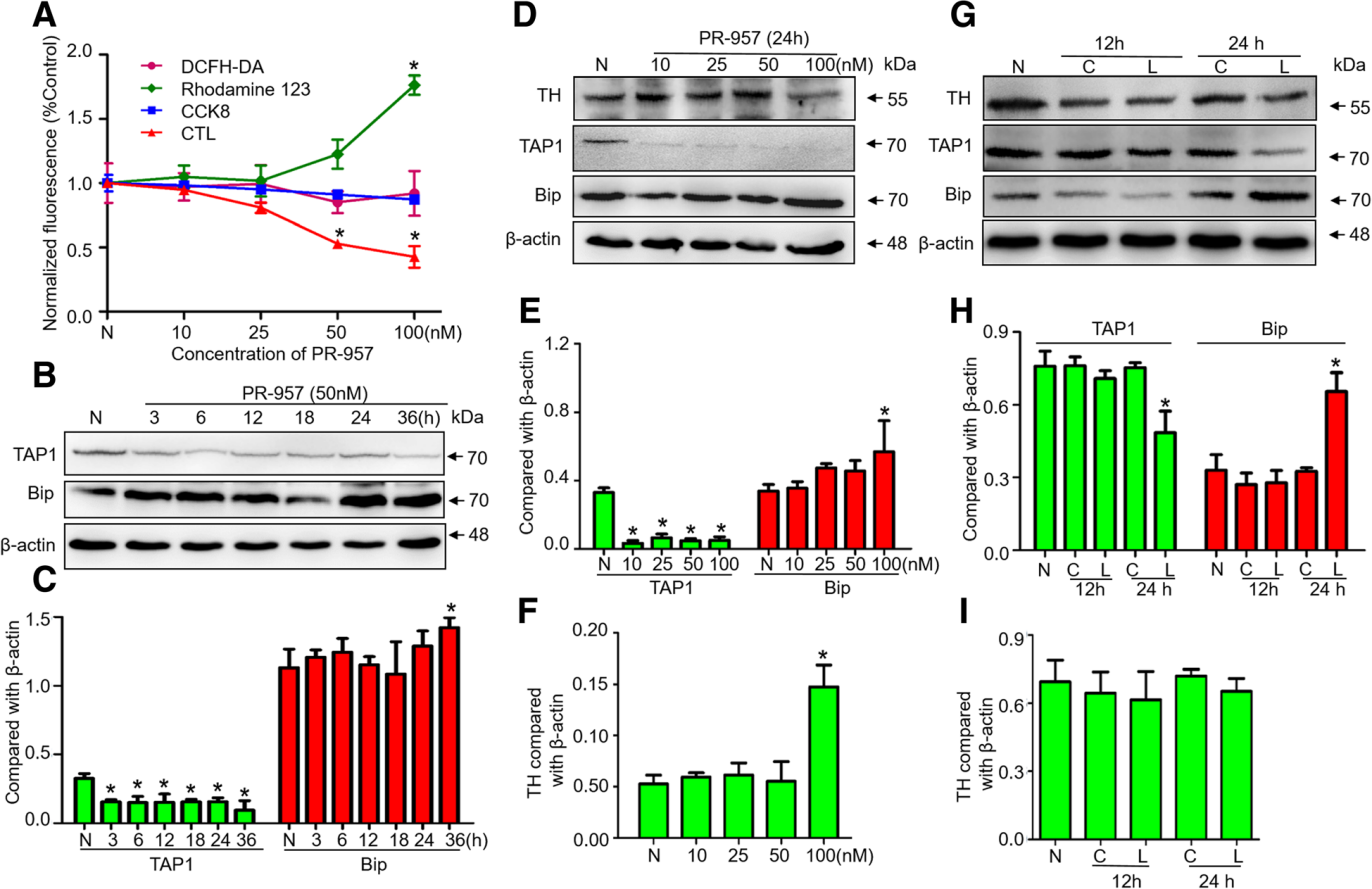
β5i inhibition impairs antigen presentation in DA neuron. **a** The cytotoxic effect and chymotrypsin-like proteasome activity in the SN4741 cell line treated with PR-957 for 24 h were estimated by various methods such as DCFH-DA, rhodamine 123, CCK8 and CTL tests (*n* = 5 experiments). DCFH-DA, rhodamine 123 and CCK-8 were respectively used to measure ROS, the mitochondrial membrane potential and cell viability. A CTL assay was used to detect the chymotrypsin-like activity of cells. (**b, c**) Western blot analysis of TAP1 and Bip in SN4741 cells after treatment with 50 nM PR-957. Western blot analysis of TH, TAP1 and Bip in SN4741 cells 24 h after PR-957 treatment (**d-f**) and in PD model rats (**g-i**). CTL, chymotrypsin-like proteasome activity. N, normal group without treatment. C, control side of the rat brain. L, lesioned side of the rat brain. Data presented as the mean ± SD; *n* = 4 experiments; * *P* < 0.05, compared with the control; one-way ANOVA and post hoc SNK t-test

SN4741 cells were then treated with 50 nM PR-957 [22]. We noted that CTL associated with β5i function decreased more than 50% but had no significant neurotoxicity in vitro (Fig. 3a). TAP1 expression was decreased after 50 nM PR-957 treatment for 3 h (Fig. 3b, c). PR-957 at 100 nM induced neurotoxicity, with significant upregulation of Bax and Bip and downregulation of TH after 24 h treatment (Fig. 3d-f). In vivo, 50 nM PR-957 was stereotaxically injected into the left anterior medial bundle in SD rats. After 24 h, TAP1 and Bip expression in the lesioned side of the SN significantly decreased and increased, respectively (Fig. 3g-h), but TH expression was not changed (Fig. 3i). Our data suggested that 50 nM PR-957 for 24 h may be optimal for β5i inhibition without significant neurotoxicity in vitro and in vivo.

### β5i inhibition exacerbates 6-hydroxydopamine-induced DA neuronal damage

**Fig. 4:**
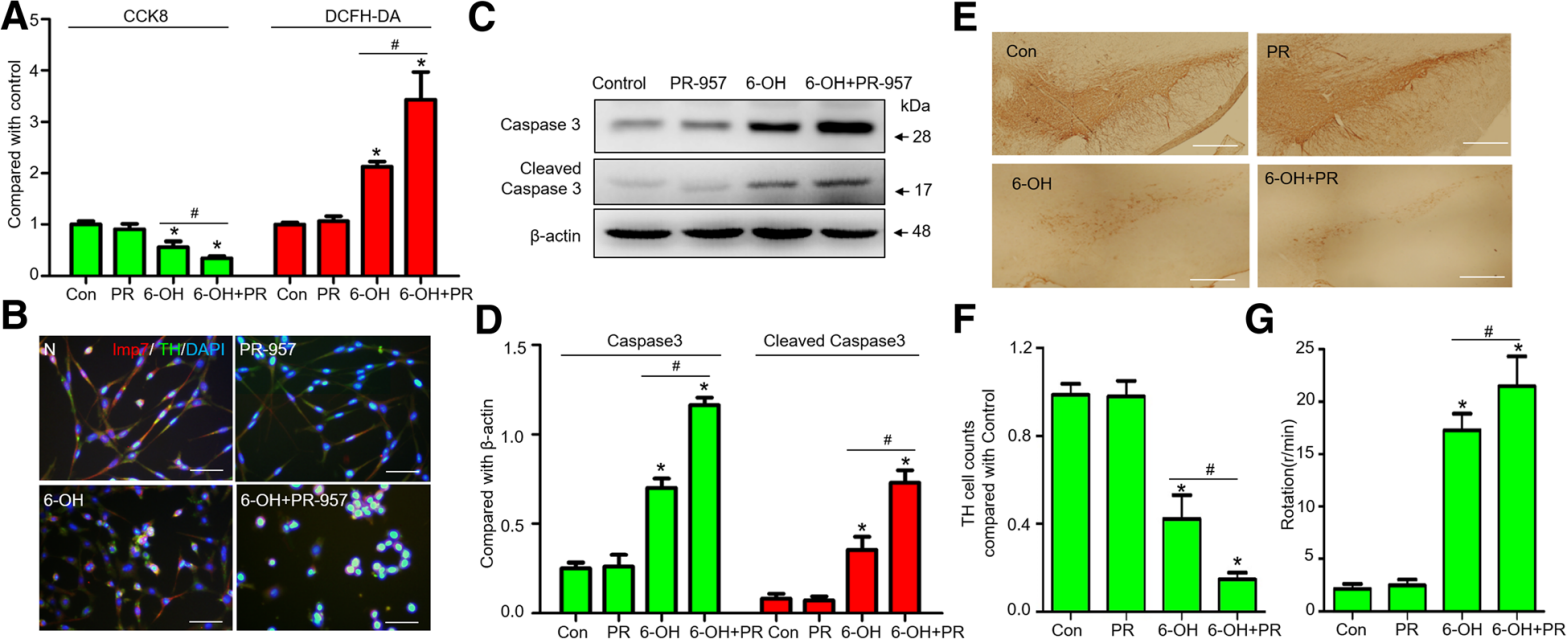
β5i inhibition exacerbates 6-hydroxydopamine-induced DA neuronal damage. **a** Cytotoxic effect on the SN4741 cell line of different treatments for 24 h analyzed by CCK8 and DCFH-DA tests (n = 5 experiments). **b** Immunofluorescence staining of β5i in SN4741 cells. Scale bar = 100 μm. **c, d** The expression of Caspase-3 and cleaved Caspase-3 in SN4741 cells after treatment with 6-OHDA or PR-957 for 24 h. **e, f** Immunohistochemistry of TH^+^ cells in rats after 6-OHDA treatment for 4 weeks. Scale bar = 200 μm. **g** Apomorphine-induced rotation scores after 6-OHDA treatment for 4 weeks (*n* = 6 experiments). Data are presented as the mean ± SD; n = 4 experiments; * P < 0.05, compared with control; ^#^ P < 0.05, compared with the 6-OHDA group; one-way ANOVA

Cell morphology, viability and apoptosis were not significantly affected by treatment with PR-957 alone in vitro. However, cotreatment with PR-957 and 6-OHDA dramatically induced cell shrinkage and pyknosis, accompanied by a significant increase in ROS and decrease in cell viability compared to treatment with 6-OHDA alone (Fig. 4a, b). Treatment with PR-957 alone did not affect Caspase-3 or cleaved Caspase-3 expression, but cotreatment significantly exacerbated the 6-OHDA-induced activation of Caspase-3 and cleaved Caspase-3 (Fig. 4c, d). In vivo, we examined the additional loss of DA neurons in the 6-OHDA hemilesioned rats after inhibition of β5i by PR-957 4 weeks after treatment. The number of TH^+^ cells in the lesioned side did not change after treatment with PR-957 alone. The 6-OHDA-induced hemilesion in the SN was partial in this 6-OHDA model. Compared to 58.3% loss of TH^+^ cells under 6-OHDA treatment alone, a 74.2% loss of TH^+^ cells was observed in the lesioned side of rats with DA neuronal damage exacerbated by PR-957, and these rats also exhibited a significant increase in apomorphine-induced rotation (Fig. 4e-g). In the striatum, the TH level on the lesioned side was only at 13.8% of that on the control side in the cotreatment group, which was significantly lower than the 21.5% in the group given 6-OHDA treatment alone (Additional file 2: Figure S2).

### Downregulated β5i expression impairs antigen presentation in DA neurons under 6-OHDA treatment

**Fig. 5:**
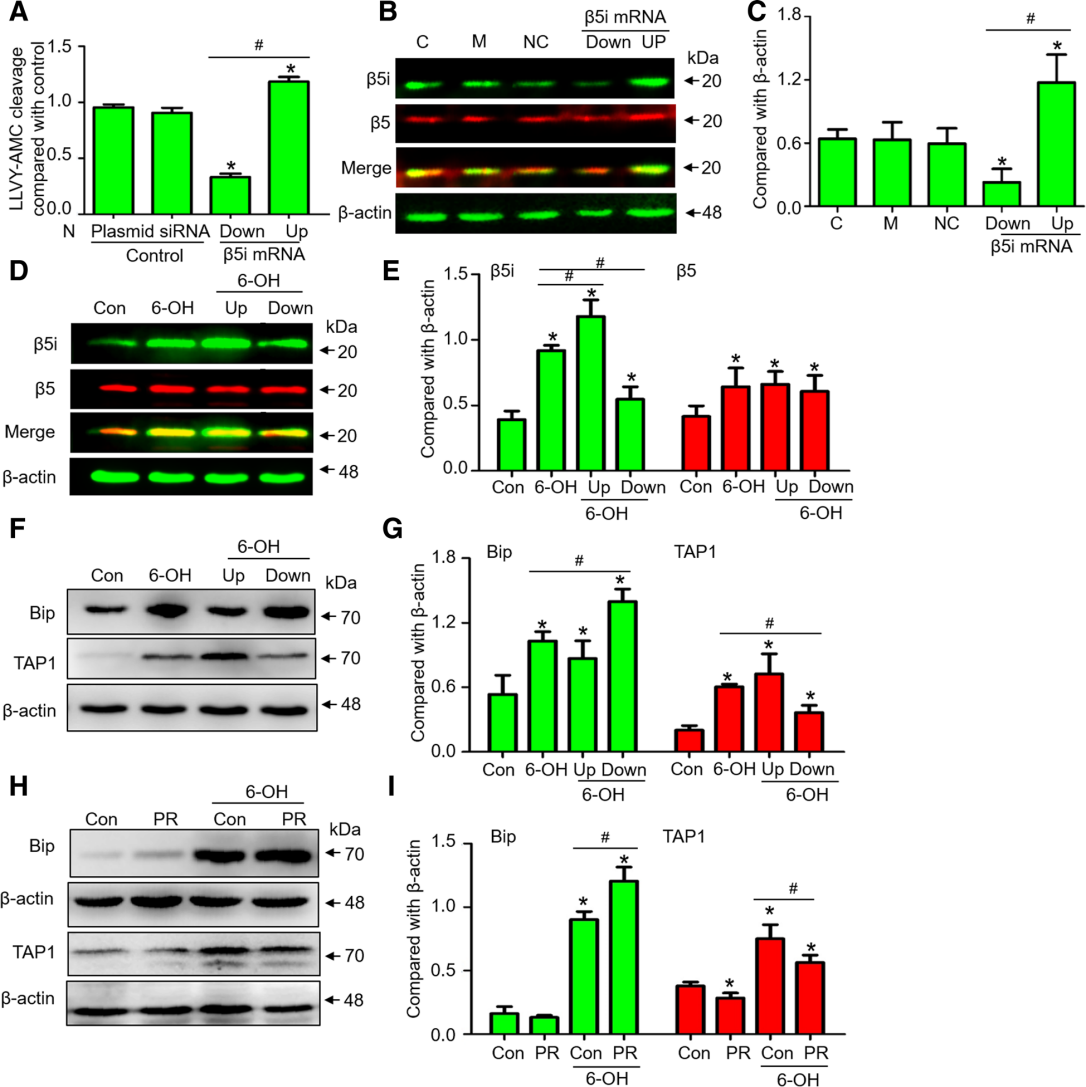
β5i regulates antigen presentation on DA neurons under 6-hydroxydopamine in vitro. **a** Chymotrypsin-like proteasome activity on SN4741 cell line was assessed by proteasome activity assays (*n* = 5 experiments). The expression of β5 and β5i in SN4741 cells at 24 h after the regulation of β5i by RNAi under normal conditions (**b, c)** and 6-OHDA treatment (**d, e**). The expression of Bip and TAP1 in SN4741 cells at 24 h after β5i downregulation by RNAi (**f, g**), and inhibition with PR-957 (**h, i**). N, normal group without treatment. C, normal condition. M, treated with an empty plasmid. NC, treated with the siRNA negative control. Up, upregulated β5i by overexpression plasmid. Down, downregulated β5i by shRNA. Data are presented as the mean ± SD; n = 4 experiments; * P < 0.05, compared with control; ^#^ *P* < 0.05, compared with the 6-OHDA group; one-way ANOVA

**Fig. 6:**
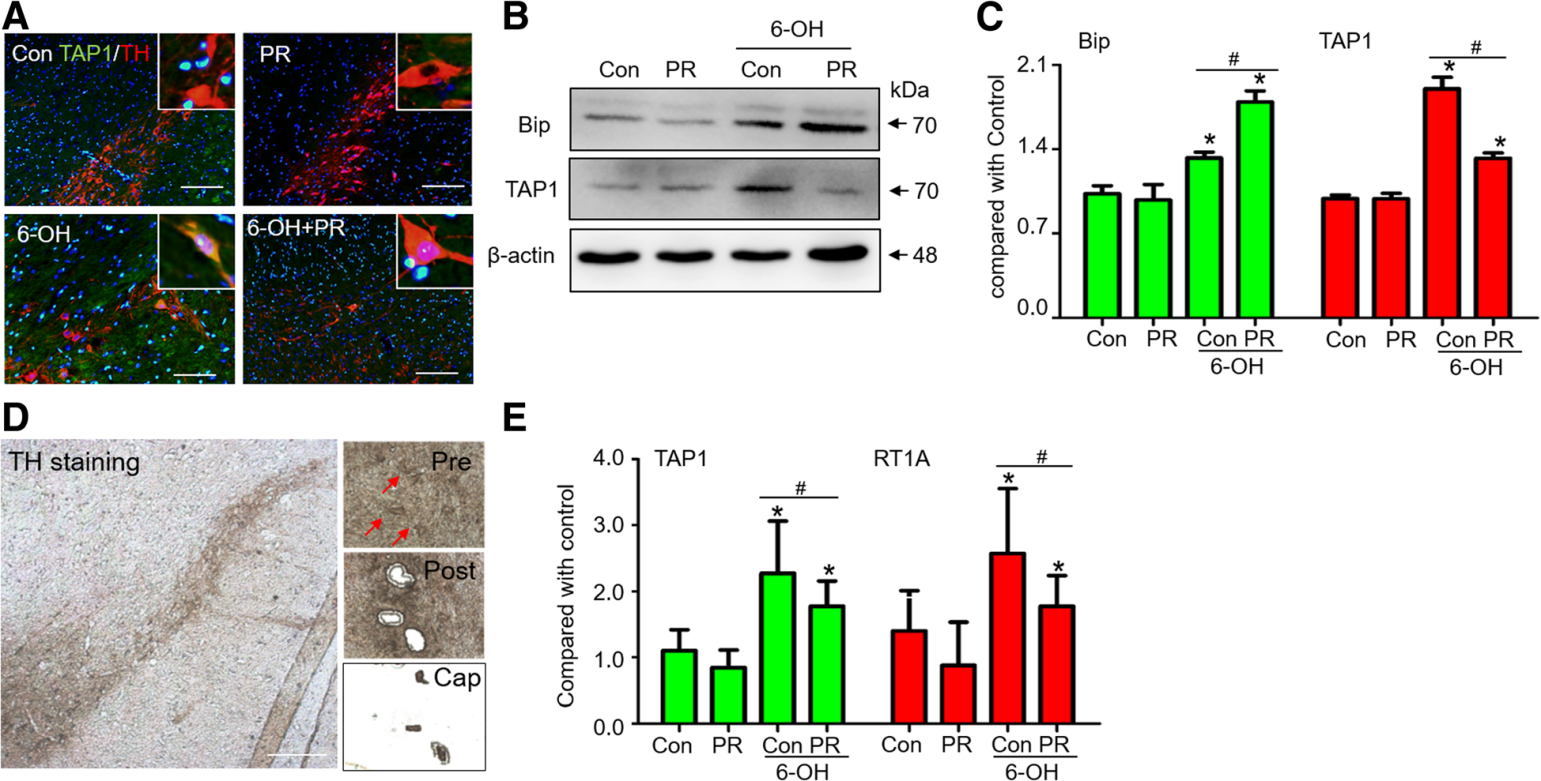
β5i regulates antigen presentation on DA neurons under 6-OHDA in vivo. **a** The expression and collocation of TAP1 after 6-OHDA for 1 day in the rat. **b, c** The expression of Bip and TAP1 at 24 h after inhibition with PR-957 in the 6-OHDA hemilesioned rats. **d** TH^+^ neurons in the SN were collected by LCM. The precaptured (Pre), postcaptured (Post) and captured (Cap) images are shown in the right column, and TH^+^ cells are indicated with red arrows. Scale bar = 100 μm. **e** qRT-PCR result of captured TH^+^ cells (*n* = 6 experiments). Error bars represent SD. * *P* < 0.05, compared with control; # *P* < 0.05, compared with 6-OHDA treatment but without PR-957

CTL function was significantly impaired and enhanced in SN4741 cells after β5i mRNA down- and upregulation, respectively (Fig. 5a). β5i expression was manipulated by RNAi in the normal condition (Fig. 5b, c) and with 6-OHDA treatment (Fig. 5d, e). In vitro, the upregulation of β5i mRNA significantly promoted TAP1 expression and inhibited Bip expression under 6-OHDA treatment, and downregulation of β5i mRNA had an inverse effect (Fig. 5f, g). After β5i function was inhibited by PR-957, the 6-OHDA-induced upregulation of TAP1 was significantly inhibited, and Bip expression was slightly increased in SN4741 cells (Fig. 5h, i) and rats (Fig. 6b, c). Immunofluorescence result showed that the 6-OHDA-induced increase in TAP1 expression was colocalized with TH^+^ cells and was attenuated by PR-957 (Fig. 6a). Laser capture microscopy was used to capture TH^+^ neurons from the SN of rats administered 6-OHDA (Fig. 6d). A significant increase in TAP1 and RT1A (MHC-I) mRNA expression in laser-captured TH^+^ neurons was found at 24 h after 6-OHDA treatment, which was inhibited by PR-957 (Fig. 6e).

## Discussion

Immunoproteasome and MHC molecules are minimally expressed in the healthy brain, and their activation and upregulation are indicative of a pathological status in the central nervous system (CNS) [12, 19, 31, 32]. The examination of brain specimens from people with neurodegenerative disorders such as AD, HD, and amyotrophic lateral sclerosis (ALS) have revealed dysfunctions in immunoproteasome activity [12, 33, 34], and these phenomena have also been found in those with autoimmune encephalomyelitis (EAE) and epilepsy [8, 15]. A similar failure in proteolytic mechanisms, as well as increases in oxidative stress and neuroinflammation, have also been reported in PD [35]. We propose the following scenario to describe the role of β5i in PD pathogenesis. Initially, ROS and the unfolded protein response (UPR) induce ER stress and neuronal damage in DA neurons. Then, Bip is activated and ships abnormal proteins from the ER to the UPS. The overloaded proteins induce the activation of β5i and transformation of the immunoproteasome to have an updated and expanded proteasome capacity. Consequently, the ubiquitinated proteins are degraded to peptides by β5i and recognized by MHC-I. Here, our study revealed that β5i is activated in DA neurons exposed to 6-OHDA, and upregulation of β5i or other immunoproteasome components might play a neuroprotective role against ROS-mediated damage in PD.

The UPS is a key factor in the proteostasis network [36]. Different subunit configurations of the UPS, such as the standard proteasome, immuneoproteasome, mixed-type proteasome, thymoproteasome and spermatoproteasome, acquire different proteolytic capacities [37]. The immunoproteasomes activated by ROS, lipopolysaccharide (LPS) and IFN-γ have strong capabilities to clear protein deposits and alleviate ROS impairment [8, 38]. The overloading of damaged proteins and insufficient proteolytic capacity may trigger immunoproteasomes to replace impaired proteasome subunits [39]. X-ray crystallography studies have shown that the enzyme active center of the immunoproteasome (iUPS) provides a larger space than the enzyme active center of other proteasomes to accommodate and degrade misfolded or oxidized proteins [40]. For example, the immunoproteasome eliminates the extended huntingtin proteins of HD, Aβ aggregates of AD and mutant SOD1 deposition of ALS more efficiently [14, 41, 42]. Under normal conditions, oxidized cytoplasmic and nuclear proteins are generally degraded by the proteasome [43]. The 20S proteasome, immunoproteasome and PA28αβ regulator are all upregulated under H_2_O_2_-induced oxidative stress, and the immunoproteasome may degrade oxidized proteins more selectively than the other proteasomes [43]. Some studies have suggested that the enhanced proteolytic activity of the immunoproteasome more efficiently clears aggregated proteins and is important for cell viability under IFN-γ treatment [8]. Others have suggested that the function of the immunoproteasome to bind and degrade ubiquitin conjugates is similar to that of constitutive proteasomes [44]. Recently, we reported that Chinese females carrying the rs17587-G/G mutation of PSMB9 are at a higher risk of PD [35]. The rs17587 variation at exon 4 of PSMB9 affects the glutamyl peptide hydrolyzing activity associated with proteolytic function [45]. As an immunoproteasome subunit, β5i has been found to be involved in proteinopathies and the innate immune response [37]. In this study, we further explored the role of β5i in the 6-OHDA model of PD. Our results showed that β5i was activated and upregulated in a dose- and time-dependent manner after 6-OHDA treatment in a DA neuron cell line, and this was further confirmed in the 6-OHDA hemilesioned rat model of PD. ER stress and oxidative stress have been suggested to contribute to the loss of DA neurons in PD [46]. Compared to the standard proteasome, the immunoproteasome is thought to be more resistant to oxidative stress and ER stress [8]. When protein homeostasis is impaired in neurons, misfolded proteins aggregate in the ER and induce ER stress [47]. Bip is upregulated and binds aggregated proteins for transportation from the ER to the UPS [29]. If the UPS and immunoproteasome system are deficient, neurons are more susceptible to apoptosis due to the stress from the accumulation of oxidized proteins [8, 48]. In aging-related sporadic inclusion body myositis, intracellular protein aggregation was accompanied by ER stress and proteasome dysfunction [49]. A study from X-linked adrenoleukodystrophy revealed that β5i was significantly elevated and recruited to mitochondria in response to oxidative stress where it participated in mitochondrial protein quality control [3]. Recently, IFN-γ-induced oxidative stress was found to upregulate β5i expression with increased poly-Ub substrate degradation efficiency [8]. In this study, we used a 6-OHDA model to induce massive oxidative stress and the unfolded protein response in DA neurons [50]. ROS and ER stress occurred in a dose- and time-dependent manner following 6-OHDA treatment [51]. We found that inhibition and downregulation of β5i resulted in DA neurons with increased sensitivity to 6-OHDA toxicity, suggesting that the neuroprotective effect of β5i may be related to ROS regulation and ER stress at the early stage of PD.

Immunoproteasomes still play an important role in the regulation of neuroinflammation [13, 52]. In the peripheral immune system, immunoproteasome subunits degrade proteins to peptides, which present to TAP1 as antigens [25]. As a peptide transporter protein, TAP1 loads antigenic peptides into the ER where MHC molecules recognize antigens and present them to the cell membrane [30, 53]. IFN-γ-signaling has been proven to promote MHC class I antigen presentation, and IFN-γ-regulated inflammation in proteasome-associated autoinflammatory syndromes (PRAAS) was partly reduced after inhibition of proteolytic function [54, 55]. As a highly selective inhibitor of β5i, PR-957 was shown to reduce the release of IL-23 and TNF-α from inflammatory cells by 90 and 50%, respectively [22]. PR-957 also inhibits inflammation in MOG35–55-induced experimental autoimmune encephalomyelitis [56]. Notably, neurodegenerative diseases predominantly display disorders of neuroinflammation. In transgenic mouse models of AD and human postmortem tissue, immunoproteasome activities and HLA-DR expression are strongly increased and accompanied by overactivated microglia in the cortex. [57, 58] Previously, neurons were considered to be ‘immunoprivileged’ without antigen presentation capabilities [59, 60]; now MHC-1 expression has been demonstrated on DA neurons in the rodent and human brain [18, 19]. The catecholaminergic neurons expressing MHC-1 have been shown to be more susceptible to apoptosis induction, suggesting that these neurons may be targeted by ROS during the development of PD [19]. In this study, our results revealed significant upregulation of MHC-I and TAP1 accompanied by increased expression of β5i on DA neurons under 6-OHDA treatment and that MHC-I and TAP1 mRNA levels were decreased after β5i inhibition. These findings suggest that β5i may regulate the TAP1/MHC-I pathway in DA neurons under oxidative stress.

## Conclusions

In conclusion, our data showed that β5i was activated by 6-OHDA-induced oxidative stress in DA neurons both in vitro and in vivo and may play a neuroprotective role in the survival of DA neurons. Our data might provide new evidence for the consideration of the immunoproteasome as a potential therapeutic target for PD.

## Additional files

Additional file 1: **Figure S1.** 6-Hydroxydopamine activates immunoproteasomes in DA neurons in a time-dependent manner. The expression of β5, β5i (A-B), TAP1 and Bip (C-D) in SN4741 cells after different durations of exposure to 200 nM 6-OHDA. * *P* < 0.05, compared with the normal condition. Data are presented as the mean ± SD; *n* = 4; one-way ANOVA and post hoc SNK t-test. (TIF 518 kb)

Additional file 2: **Figure S2.** β5i inhibition exacerbates 6-hydroxydopamine-induced damage in the striatum. (A) Immunostaining of TH in the rat striatum after 6-OHDA treatment for 4 weeks. Scale bar = 200 μm. (B) Quantification of TH immunoreactivity in the striatum. Data are presented as the mean ± SD; n = 4 experiments; * *P* < 0.05, compared with the control; # *P* < 0.05, compared with the 6-OHDA group; one-way ANOVA. (TIF 1137 kb)
