# Supplementary figures and images for: Dopaminergic neurons show increased low-molecular-mass protein 7 activity induced by 6-hydroxydopamine in vitro and in vivo
Source: Transl Neurodegener. 2018 Aug 17;7:19. doi: 10.1186/s40035-018-0125-9 (PMC6097308; doi:10.1186/s40035-018-0125-9)

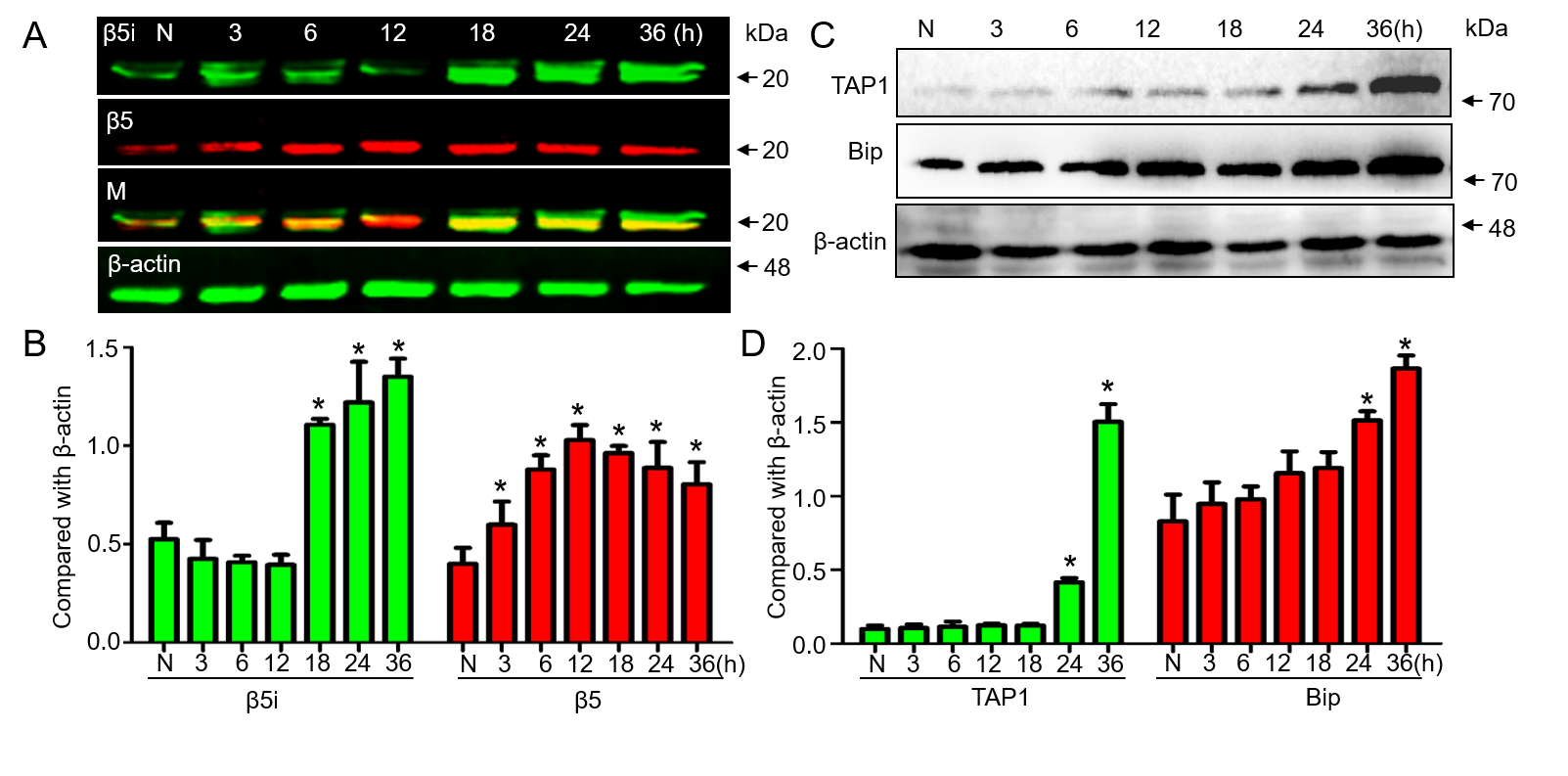

Supplement: Supplementary file 1 — Figure S1. 6-Hydroxydopamine activates immunoproteasomes in DA neurons in a time-dependent manner. The expression of β5, β5i (A-B), TAP1 and Bip (C-D) in SN4741 cells after different durations of exposure to 200 nM 6-OHDA. * P < 0.05, compared with the normal condition. Data are presented as the mean ± SD; n = 4; one-way ANOVA and post hoc SNK t-test. (TIF 518 kb) [file 40035_2018_125_MOESM1_ESM.tif]

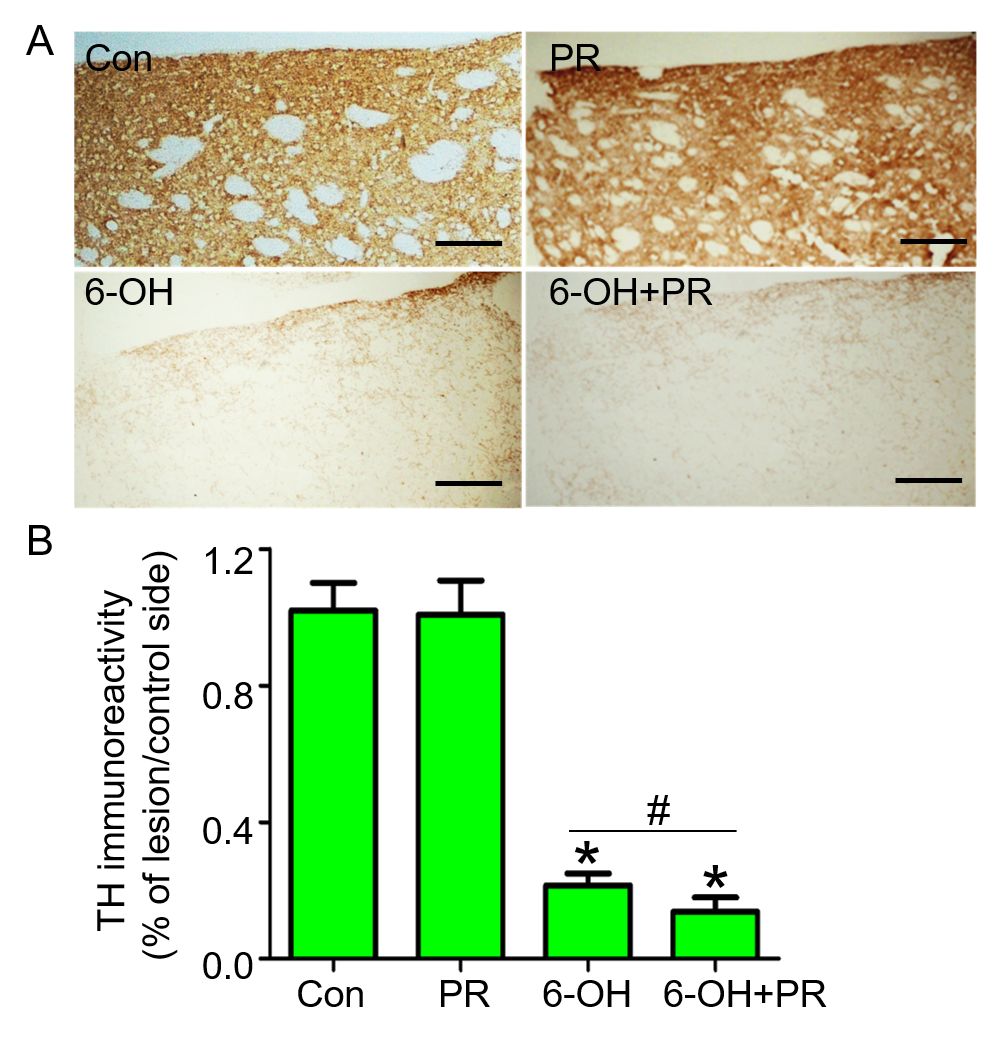

Supplement: Supplementary file 2 — Figure S2. β5i inhibition exacerbates 6-hydroxydopamine-induced damage in the striatum. (A) Immunostaining of TH in the rat striatum after 6-OHDA treatment for 4 weeks. Scale bar = 200 μm. (B) Quantification of TH immunoreactivity in the striatum. Data are presented as the mean ± SD; n = 4 experiments; * P < 0.05, compared with the control; # P < 0.05, compared with the 6-OHDA group; one-way ANOVA. (TIF 1137 kb) [file 40035_2018_125_MOESM2_ESM.tif]
